# Prognostic analysis of lymph node ratio of patients with disease recurrence previously submitted to cervical dissection surgery for head and neck cancer

**DOI:** 10.1590/0100-6991e-20223178-en

**Published:** 2022-05-20

**Authors:** JOÃO PAULO ZENUN RAMOS, FELIPE RAULE MACHADO, VANIA APARECIDA LEANDRO MERHI, JOSÉ LUÍS BRAGA DE AQUINO

**Affiliations:** 1 - Pontifícia Universidade Católica de Campinas, Cirurgia de Cabeça e Pescoço - Campinas - SP - Brasil

**Keywords:** Head and Neck Neoplasms, Neck Dissection, Lymph Node Ratio, Neoplasias de Cabeça e Pescoço, Esvaziamento Cervical, Razão entre Linfonodos

## Abstract

**Introduction:**

the variable lymph node ratio has recently been studied as a possible influencer in the survival of patients diagnosed with head and neck cancer.

**Objective:**

to analyze the correlation between lymph node density and survival of recurred disease patients previously submitted to cervical dissection surgery due to head and neck squamous cell carcinoma.

**Method:**

we retrospectively analyzed 71 medical records of patients treated at the Head and Neck Surgery Service of the Pontifícia Universidade Católica de Campinas who had undergone cervical dissection surgery and presented tumor recurrence between 2006 and 2019. Patient and tumor data such as age, gender, skin color, smoking, alcohol consumption, location of the primary tumor, anatomopathological characteristics and lymph node status were correlated with the survival time.

**Results:**

we found a predominance of males and the mean age was 59.5 years. The most frequent primary site was the oral cavity followed by the larynx and oropharynx. The mortality rate was 53.52% and the mean lymph node ratio 0.28. We found influence on survival with statistical significance for the parameters: lymph node ratio, number of dissected and affected lymph nodes, T and N staging, type of treatment proposed (palliative or surgical), presence of compromited margins in the primary tumor and lymph node extravasation.

**Conclusion:**

the calculation of lymph node density in patients with recurred disease after cervical dissection surgery by head and neck squamous cell carcinoma should be taken into account during therapeutic planning and prognostic evaluation due to its direct influence on the survival.

## RESUMO

**Introdução:**

recentemente tem-se estudado a variável densidade linfonodal como possível influenciador na sobrevida de pacientes com diagnóstico de câncer de cabeça e pescoço.

**Objetivo:**

analisar a relação entre a densidade linfonodal e a sobrevida de pacientes recidivados previamente submetidos a cirurgia de esvaziamento cervical por carcinoma espinocelular de cabeça e pescoço.

**Método:**

foram analisados retrospectivamente 71 prontuários pacientes atendidos no Serviço de Cirurgia de Cabeça e Pescoço da Pontifícia Universidade Católica de Campinas que tinham sido submetidos a cirurgia de esvaziamento cervical e apresentaram recidiva tumoral entre os anos de 2006 e 2019. Dados relativos ao paciente e ao tumor tais como: idade, sexo, cor da pele, tabagismo, etilismo, localização do tumor primário, características anatomopatológicas e status linfonodal foram correlacionados ao tempo de sobrevida dos indivíduos.

**Resultados:**

encontramos predominância do sexo masculino e a média de idade foi de 59,5 anos. O sítio primário mais frequente foi a cavidade oral seguido da laringe e orofaringe. A taxa de mortalidade foi de 53,52% e a densidade linfonodal média 0,28. Encontramos influência na sobrevida com significância estatística para os parâmetros: densidade linfonodal, número de linfonodos dissecados e acometidos, estadiamento T e N, tipo de tratamento proposto (paliativo ou cirúrgico), presença de margens comprometidas no tumor primário e extravasamento linfonodal.

**Conclusão:**

o cálculo da densidade linfonodal em pacientes recidivados após cirurgia de esvaziamento cervical por carcinoma espinocelular de cabeça e pescoço deve ser levado em consideração durante o planejamento terapêutico e na avaliação prognóstica devido à sua direta influencia na sobrevida dos indivíduos.

## INTRODUCTION

Approximately 500 thousand new cases of head and neck Squamous Cell Carcinoma (SCC) are diagnosed each year in the world and their incidence represents about 3% of all cancers^1^.

About 40% of Head and Neck Neoplasms (HNN) are located in the oral cavity, followed by the oropharynx and larynx. The most frequent histological type is the SCC, present in more than 90% of cases^2^.

In Brazil no precise statistics are available; however, considering the oral cavity SCC, it is estimated that the number of new cases of this disease expected in Brazil for each year in the 2020-2022 triennium will be 11,180 cases in men and 4,010 in women, ranking fifth among all cancers in males and thirteenth in females^3^. 

Although this neoplasm affects mostly men, in recent years there has been a notable prevalence increase among women. There are several risk factors associated with head and neck cancer, such as socioeconomic status, diet, oral hygiene, occupational exposure, family history and even some infections; however, the most important known risk factors are smoking and alcohol abuse which, when associated, boost the risk of carcinogenesis^4^ ^-^ ^6^. 

The staging system used worldwide for this disease is the one recommended by the Union for International Cancer Control (UICC), called the TNM system for the classification of malignant tumors (UICC) and the treatment depends on factors such as the tumor location, clinical staging as well as the patient’s physical conditions. Treatment can include surgery, radiotherapy and/or chemotherapy, separately or in combination. However, tumors with the same TNM staging may demonstrate different patterns of evolution, suggesting the need for assessing other factors capable of more accurately determining disease prognosis.

The main route of dissemination of head and neck SCC is lymphatic. Therefore, pathological neck condition is one of the determining factors for indicating adjuvant therapy^7^.

The concept of Lymph Node Density (LND), represented by a numerical ratio between the number of lymph nodes affected by the neoplasm, confirmed by an anatomopathological assessment, over the total number of lymph nodes surgically resected, has already been described and is used in oncology clinic in breast, esophagus, stomach, cardia and rectum tumors for example^8^ ^-^ ^12^; however, since Shrime et al.^13^ published their first article suggesting the application of this parameter in head and neck surgery, the possibility of LND being an independent prognostic factor in relation to head and neck patients surviving SCC has been discussed^14^ ^,^ ^15^. In addition, some studies compare the prognostic efficacy of LND with the actual TNM classification, showing different results^16^ ^-^ ^19^.

Thus, the objective of this study is to assess whether lymph node density had an impact on the survival of patients who had previously undergone cervical dissection surgery due to SCC of the oral cavity, larynx or pharynx.

## METHODS

This is a longitudinal retrospective study carried out by reviewing the medical records of patients at the Head and Neck Surgery outpatient clinic of an university hospital who underwent cervical dissection due to SCC of the oral cavity, larynx or pharynx between 2006 and 2019 and experienced recurrence of the disease. Patients with other histological types or who had already undergone chemotherapy or radiation therapy prior to the first surgery were excluded.

In addition to the descriptive analysis of the sample, we studied the factors associated with the time of death using Cox Regression. In the continuous variables such as age, number of resected lymph nodes, number of lymph nodes affected and lymph node density, the Maximally Selected Rank Statistics method was used to find the cut-off points that differed most to serve as a comparative parameter.

We also evaluated the influence of other variables on survival, such as gender, skin color, age, smoking abuse, alcohol abuse, anatomical site of the primary tumor, adjuvant treatment after the first surgery, location of recurrence, type of treatment proposed after recurrence, number of resected lymph nodes and affected lymph nodes, T and N staging, status of the specimen margins, presence of perilymphatic and/or perineural invasion and extranodal extension.

This investigation was approved by the research ethics committee of the Pontifical Catholic University of Campinas (PUC-Campinas), under Opinion number 3,480,409.

## RESULTS

We selected 71 patients, with a mean age of 59.5 years. There was a predominance of males (76.0%), more than 42.0% of individuals were brown, 38% white and 19.7% black. The great majority was of smokers (91.5%) and alcoholics (61.9%).

The most frequent location of the primary tumor was the oral cavity (43.6%), followed by the larynx (29.5%) and oropharynx (26.6%). The most frequent T staging was T3 (36.6%), followed by T4, T2 and T1. Regarding the N staging, the absolute majority (76.0%) was in N2 stage. The study of the primary tumor margins showed that 57.7% were free, 26.7% small (up to 0.5cm) and 15.4% affected. Lymphatic dissemination was identified in 81.6% of the patients and perineural in 77.4% of surgical specimens. Extranodal extension was observed in 43.6% of the individuals.

After the first surgery, adjuvant therapy was performed and 84.5% of the patients underwent radiotherapy and 66.3% chemotherapy. The main site of recurrence was the primary site with 73.2%. Most patients (69.0%) underwent rescue surgery after a recurrence was diagnosed. In total, thirty-eight patients (53.5%) died. Currently, 29 patients are undergoing outpatient follow-up without evidence of active disease and 9 are undergoing oncological follow-up with palliative care.

In the assessment of the lymph node status, the number of lymph nodes dissected varied between 14 and 175, with an average of 50.4 lymph nodes per dissection. Regarding the number of affected lymph nodes, we found a value between 1 and 41 with a mean of 15.4 and the lymph node density ranged from 0.05 to 0.68 with a mean of 0.28. The patients’ mean survival time was 74.4 months and the 1, 2, 3, 4 and 5 year survival was estimated at 83.00%, 72.96%, 58.00%, 53.00% and 51.20%, respectively.

The best cutoff points that differentiated the time of death for age, number of resected lymph nodes, number of affected lymph nodes and lymph node density were, respectively, 67 years, 49, 15 and 0.18.

We used the Cox Regression to assess which variables influenced the risk of death. We found statistical significance in the factors: number of affected lymph nodes, number of lymph nodes dissected, lymph node density, T stage, N stage, type of treatment proposed after the diagnosis of recurrence, quality of the primary tumor margins and extranodal extension. In the other variables studied, there was no statistical significance. Table 1 summarizes the relative risk of each variable studied, in addition to explaining the comparison of survival between 1 and 5 years.

**Table 1:** Cox regression to assess factors associated with death (results that were statistically significant).

Variables	Effect vs reference	p-value	RR	95%CI RR	1 year survival	5 years survival
Number of lymph nodes affected	≥15 vs <15	<0.001	4.73	2.44-9.19	62.5 vs 93.4	16.7 vs 67.2
Resected lymph nodes	≥49 vs <49	0.005	2.60	1.33-5.10	73.0 vs 93.9	38.6 vs 64.8
Lymph nodes density	≥0.18 vs <0.18	<0.001	4.41	2.12-9.16	70.1 vs 97.0	27.8 vs 74.7
T	T3 vs T2	0.071	2.43	0.93-6.38	84.6 vs 100.0	49.1 vs 69.1
	T4 vs T2	0.005	3.89	1.50-10.08	66.7 vs 100.0	34.3 vs 69.1
N	N2A vs N1	0.817	1.16	0.32-4.17	88.2 vs 100.0	69.5 vs 68.6
	N2B vs N1	0.540	1.46	0.44-4.86	100.0 vs 100.0	54.9 vs 68.6
	N2C vs N1	0.024	3.61	1.19-10.99	75.0 vs 100.0	31.2 vs 68.6
Proposed treatment	Palliative vs Surgical	<0.001	3.27	1.70-6.27	68.2 vs 89.7	27.3 vs 62.6
Margins	Committed vs Free	0.003	3.45	1.52-7.82	54.5 vs 90.2	27.3 vs 65.1
	Small vs Free	0.037	2.22	1.05-4.69	83.9 vs 90.2	34.2 vs 65.1
Extranodal Extension	Yes vs No	<0.001	4.02	2.06-7.85	67.7 vs 94.9	22.9 vs 72.7

*Note: Bold numbers represent results that were statistically significant.*

Abbreviations: CI: confidence interval; N: staging; RR: relative risk; T: staging.

**Figure 1:**
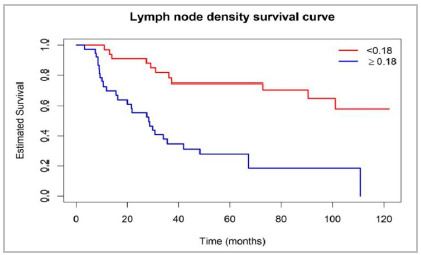
Cox regression to assess factors associated with death (results that were statistically significant).

We can see in Figure 1, the Kaplan-Meier curve that illustrates the difference in survival between patients who had LND values greater than or equal to and less than 0.18.

## DISCUSSION

Our study is characterized by the fact that the sample studied is composed exclusively of patients who had already undergone CE and yet experienced recurrence of the disease. Thus, we have a more homogeneous group for data analysis.

Although we are located in one of the most developed urban regions in the country, we often receive patients referred from different States. Therefore, we found results consistent with the Brazilian literature, especially regarding the demographic profile of patients^2^. The unfavorable prognosis for patients with more advanced stages such as III and IV has been described in the literature for some years^20^ ^,^ ^21^. 

In relation to N staging, we compared the survival of N1 patients with those of N2a, N2b and N2c and we found statistical significance with worse survival of N2c compared to N1 patients.

Regarding patients N2a and N2b, there was no statistical significant difference. We can therefore infer that the laterality factor may have had an influence in this case, since the main characteristic of the N2c is neck with counter or bilaterality metastases. According to Ferlito et al.^22^, the presence of the contralateral lymph node, whether accompanied by ipsilateral cervical involvement or not, was shown to be an influencing factor in decreasing the survival of these individuals. 

Still discussing the parameters related to the tumor, we identified with statistical significance that extracapsular extravasation had an influence on the survival of individuals. This finding was in agreement with what the literature has currently presented, a fact that contributed to the incorporation of this parameter in the new TNM staging system 8th edition of the UICC^23^.

Another widely used oncology parameter is the presence of margins that are affected or not after resection of the primary lesion^24^. Our study had a methodological advantage, i.e. it was carried out entirely with a sample of patients treated in the same service, with the same team of surgeons and pathologists, which, in a way, generates greater reliability due to the standardization of both the surgical technique and the anatomopathological evaluation. We showed with statistical significance that patients with affected tumor surgical margins had a shorter survival than those who had free margins.

Regarding LND, there is still no consensus in the literature as to what would be the ideal cutoff density value that influences the survival of individuals. Prabhu et al.^25^ evaluated patients with oral cavity and larynx SCC, and showed that lymph node density above 0.2 implied in decreased survival; in addition, it was calculated that with each 1% increase in the LND value, the risk of disease recurrence and death increased 1.02 times. Recently Talmi et al.^26^ concluded that the lymph node density reported in the world literature that has an influence on survival ranged from 0.02 to 0.2 with an average of 0.09^26^ ^,^ ^27^. 

The total value of the resected lymph nodes during dissection is extremely important in order to be able to have confidence from the perspective of surgical oncology. Some studies have used the total value of resected lymph nodes to study whether this parameter is an independent prognostic variable^28^. A criticism regarding the use of isolated lymph node density as an independent prognostic variable is precisely due to the fact that it is a simple numerical relationship. Therefore, we must also have access to the total number of lymph nodes resected during cervical dissection so that the lymph node density value can be valid. In our study, we had a considerably high number of lymph nodes resected by dissections (average of 50.4) most likely due to the greater number of radical than selective dissection, since we evaluated cases of greater severity.

Several authors have tried to evaluate the ideal number of lymph nodes resected in an EC in order to consider a reliable staging^29^ ^,^ ^30^. A recent systematic review found between 11 to 25 lymph nodes resected with an average of 18, so that the higher the number, the greater the survival of patients^28^. Divi et al.^7^ ,after a large study involving an American database, also concluded that the minimum number of lymph nodes resected for adequate neck dissection is 18, and patients with a lower number of lymph nodes resected had a higher risk of death. In a recent publication, UICC considers the minimum number of 16 lymph nodes^31^. 

Some studies argue that the LND may suggest a better survival forecast than the actual TNM classification, while others suggest that this variable, in some cases, can be used as a tool when indicating an adjuvant therapy such as chemo or radiotherapy^30^ ^,^ ^32^ ^-^ ^34^.

In our study, we found a considerably high number of both resected and affected lymph nodes, and also lymph node density was higher than the average found in the literature^26^. This result is probably justified by the fact that the sample of patients evaluated in our study was composed exclusively of relapsed patients.

We believe that the simple application of TNM staging to define clinical and/or surgical procedures in patients with head and neck SCC may be insufficient. As we demonstrated in this study, there are several variables that should not be ignored for a complete assessment of each case. Intrinsic characteristics of individuals and anatomical specimens that are not included in the UICC TNM staging can and should be considered to minimize the indication of excessively morbid or even insufficient therapies from the standpoint of head and neck oncology.

## CONCLUSION

We conclude that the lymph node density of recurrent patients previously submitted to cervical dissection surgery due to head and neck SCC had an influence on the individuals’ survival, so that higher values of lymph node density are related to lower survival. In addition, both the total number of lymph nodes surgically resected and the absolute number of lymph nodes affected by the neoplasm had also an influence on the prognosis. The intrinsic characteristics of the surgical specimen, such as the size of the primary tumor, the N staging, the quality of the margins and the presence of extracapsular extravasation, also proved to be important variables for a more accurate post-surgical staging, since, in our study, all these parameters showed an influence on survival with statistical significance.
